# Disentangling stable traits from dynamic processes: a longitudinal investigation of academic burnout and problematic mobile phone use

**DOI:** 10.3389/fpsyt.2026.1873653

**Published:** 2026-07-22

**Authors:** Guanghui Shen, Yunong Han, Sheng Xu, Lili Chen, Xiuhong Xu, Xinyu Hu, Zixin Ye, Li Chen, Yu Li

**Affiliations:** 1 Department of Child Psychiatry, Wenzhou Seventh People’s Hospital, Wenzhou, China; 2 School of Mental Health, Wenzhou Medical University, Wenzhou, Zhejiang, China; 3 Department of Pediatrics, The Second Hospital of Hebei Medical University, Shijiazhuang, China; 4 Department of Pediatrics, The Second Affiliated Hospital and Yuying Children’s Hospital of Wenzhou Medical University, Wenzhou, China; 5 Department of Teacher Education, Lishui University, Lishui, Zhejiang, China; 6 Mental Health Education Center of School of Marxism, Zhejiang Gongshang University, Hangzhou, Zhejiang, China

**Keywords:** academic burnout, longitudinal, longitudinal study, problematic mobile phone use, university freshmen

## Abstract

**Background:**

Problematic mobile phone use (PMPU) has become a prevalent behavioral concern among university students, particularly in China. Although academic burnout is considered a key stressor, the temporal directionality between burnout and PMPU remains inconclusive. This study aimed to clarify their longitudinal relationship using a Random Intercept Cross-Lagged Panel Model (RI-CLPM).

**Methods:**

A three-wave longitudinal design was employed among Chinese university freshmen (N = 4,903; mean age = 19.28 years; 59.7% female), with data collected at six-month intervals over one year. Academic burnout and PMPU were assessed using validated self-report scales. RI-CLPM was applied to disentangle between-person and within-person effects. Measurement invariance and gender invariance were also tested.

**Result:**

At the between-person level, academic burnout was positively associated with PMPU (*β* = 0.34, *p* < 0.001), indicating stable trait-level co-occurrence. At the within-person level, increases in academic burnout significantly predicted subsequent increases in PMPU (*β* = 0.07, *p* = 0.01). However, the reverse effect from PMPU to academic burnout was not significant (*β* = 0.03, *p* = 0.08). These findings support a unidirectional relationship. Multi-group analyses further demonstrated that the model was invariant across gender (Δ*χ²* = 2.11, *p* = 0.72).

**Conclusion:**

The findings provide longitudinal evidence that higher levels of academic burnout prospectively predict subsequent increases in PMPU. PMPU may reflect a maladaptive coping response to academic resource depletion. These findings suggest that interventions targeting academic burnout may help reduce subsequent risk for PMPU.

## 1 Introduction

With the rapid proliferation of mobile technology, mobile phones have become deeply embedded in students’ daily communication, information seeking, learning, and entertainment. However, maladaptive overuse of these devices has become a growing public health concern (1). Problematic mobile phone use (PMPU) refers to a pattern of poorly regulated use characterized by craving, withdrawal-like experiences, impaired control, and functional impairment, and it is often discussed within the broader framework of behavioral addiction (2). Epidemiological evidence suggests that PMPU is particularly common among university students in China, with reported prevalence estimates ranging from 21.3% to nearly half of undergraduate samples (3, 4). This pattern is concerning because PMPU has been associated with adverse developmental outcomes, including compromised academic performance (5), sleep disturbances (6), and elevated risks of psychopathology such as depression and anxiety (7). Identifying factors that may precede changes in PMPU is therefore important for prevention and intervention. Although previous studies have examined relatively stable individual characteristics, such as neuroticism and impulsivity, as risk factors (8), these trait-level explanations do not fully account for why PMPU may fluctuate across the university transition. Situational academic stressors, particularly academic burnout, may provide a more dynamic explanation for changes in mobile phone overuse during this period. A key unresolved issue is whether academic burnout merely co-occurs with PMPU because some students are generally more vulnerable to both problems, or whether increases in burnout within the same student prospectively precede later increases in PMPU.

### 1.1 The theoretical mechanism: resource depletion and compensatory coping

Academic burnout, originally conceptualized in occupational research, refers in students to exhaustion from study demands, cynical or detached attitudes toward schoolwork, and a reduced sense of academic efficacy (9). The association between academic burnout and PMPU can be understood by integrating Conservation of Resources (COR) theory (10) with the Compensatory Internet Use Theory (CIUT) (11). According to COR theory, individuals strive to obtain, protect, and restore valued resources. When resource loss is experienced or anticipated, individuals may seek strategies that reduce further depletion or provide short-term recovery (12). CIUT helps explain why mobile phone use may become one such strategy. Students experiencing burnout may have limited energy for active, problem-focused coping (13). Mobile phones provide an immediately available, low-effort, and immersive route to distraction, social contact, and temporary relief+.

A. Over time, however, repeated reliance on this coping route may be associated with poorer self-regulation and more severe PMPU symptoms. Thus, PMPU is best understood in the present study as a potential maladaptive coping response linked to academic resource depletion, rather than as a direct or inevitable consequence of burnout.

### 1.2 The empirical status: inconsistent evidence and methodological evolution

Although the theoretical link between academic burnout and PMPU is plausible, empirical evidence regarding their temporal ordering remains mixed. Cross-sectional studies have consistently reported a positive association between academic burnout and PMPU (14, 15). but such designs cannot clarify whether burnout tends to precede PMPU, PMPU tends to precede burnout, or both processes unfold reciprocally. Existing longitudinal studies have also produced inconsistent patterns. Some findings are compatible with a burnout-to-PMPU pathway (2, 16)., whereas others suggest that excessive mobile phone use may displace study time and contribute to later burnout (17), or that the two constructs may reinforce each other over time (18).

These inconsistencies may largely stem from a methodological limitation prevalent in prior literature: the reliance on traditional Cross-Lagged Panel Models (CLPM) (19). Although widely utilized, the traditional CLPM has been criticized for failing to distinguish between-person differences (stable, trait-like characteristics) from within-person fluctuations (state-like deviations) (20). By conflating these two distinct sources of variance, CLPMs often produce biased estimates, where observed causal effects may actually reflect stable trait correlations rather than genuine dynamic processes (21). To rigorously clarify the mechanism, it is imperative to move beyond traditional appoaches and employ the Random Intercept Cross-Lagged Panel Model (RI-CLPM). By explicitly disentangling stable traits from state-dependent changes, the RI-CLPM allows for a precise examination of how fluctuations in academic burnout drive changes in PMPU at the individual level, providing a more robust test of the underlying causality (22).

### 1.3 The present study: contextual focus and gender invariance

**Figure 1:**
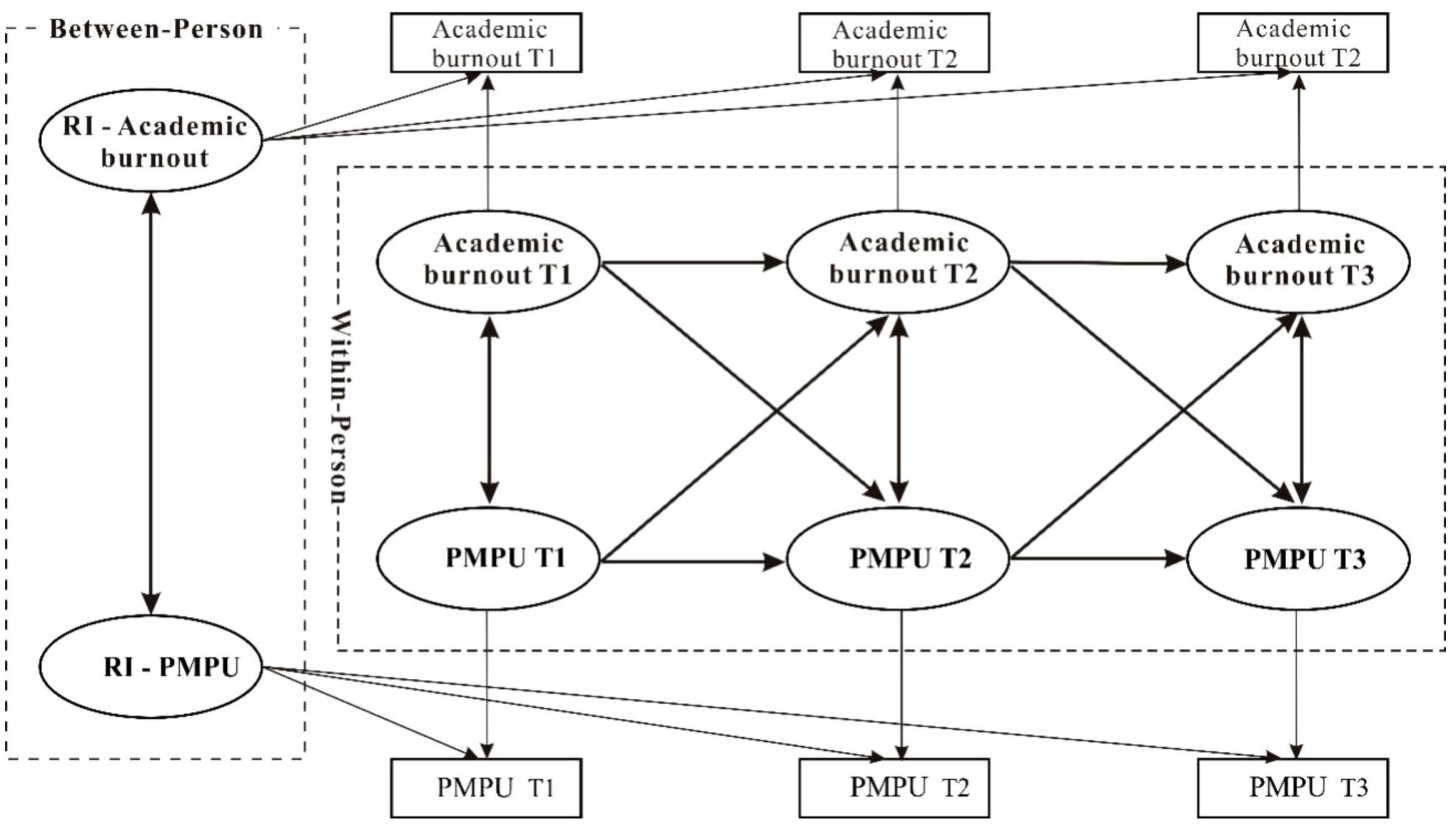
The hypothesized random intercept cross-lagged panel model (RI-CLPM). PMPU, Problematic mobile phone use; T1–T3, Time 1 to Time 3.

To address these theoretical and methodological gaps, the present study adopted a three-wave longitudinal design to investigate the bidirectional relationship between academic burnout and PMPU Chinese freshmen, a cohort experiencing a critical window of resource fluctuation following the competitive college entrance examination (Gaokao) (23). This transitional context is ideal for examining the dynamic interplay between stress and maladaptive coping (24). In addition, because previous studies have suggested possible gender differences in mobile phone use patterns and digital coping, we tested whether the longitudinal structure of the model was invariant across gender. Specifically, utilizing the RI-CLPM, the hypothesized model is depicted in **Figure 1**: (H1) at the between-person level, academic burnout will be positively associated with PMPU, reflecting stable individual differences shared by both constructs; (H2) at the within-person level, increases in academic burnout will predict subsequent increases in PMPU over time; and (H3) the longitudinal relationships between burnout and PMPU will be invariant across gender, suggesting a common underlying mechanism for both male and female students.

## 2 Methods

### 2.1 Participant and procedure

Participants were recruited from four universities in Zhejiang Province, China, using convenience sampling. The inclusion criteria were: (1) being a full-time freshman student; (2) agreeing to participate in the study and signing the informed consent form; and (3) passing the embedded attention check items. The study employed a three-wave longitudinal design over the course of one year, with assessments conducted at six-month intervals: Wave 1 (T1) in September 2023 (freshman entry), Wave 2 (T2) in March 2024, and Wave 3 (T3) in September 2024. Of the initial sample of 5,617 completed the T1 assessment and 4,903 completed the T3 assessment, representing a 87.29% retention rate. The final sample consisted of 1,977 males and 2,926 females, with a mean age of 19.28 years (*SD* = 0.67).

Data collection was conducted in university computer laboratories across three waves. Trained research assistants administered the computer-based surveys using standardized protocols. To ensure confidentiality, participant responses were matched longitudinally using anonymized unique identifiers. Following each assessment, participants received a personalized feedback report. The study protocol was approved by the Ethics Committee of the author University (No. 2023021) and adhered to the Declaration of Helsinki. Written informed consent, including the right to withdraw, was obtained from all participants.

### 2.2 Measures

#### 2.2.1 Demographic information

Demographic information was collected using a self-report questionnaire. Participants will be asked to provide their age in years, gender (coded as male = 0, female = 1).

#### 2.2.2 Problematic mobile phone use

PMPU was assessed using the Mobile Phone Addiction Index (MPAI) developed by Leung (25). The MPAI contains 17 items with a 5-point Likert scale ranging from 1 (not at all) to 5 (always). Total scores range from 17 to 85. Previous studies with university student samples have reported the MPAI to possess robust internal consistency and construct validity(Cronbach’s α= 0.86) (26). In this study, Cronbach’s alpha coefficients for the MPAI across the three measurement points (T1 through T3) were found to be 0.90, 0.92, and 0.94, respectively. Furthermore, the MacDonald ω coefficients range from 0.91 to 0.94 across T1 to T3.

#### 2.2.3 Academic burnout

The Chinese version of the Learning Burnout Scale (LBS) was used to assess academic burnout. The LBS has been widely used among Chinese student populations and has demonstrated good internal consistency in previous research, with Cronbach’s alpha coefficients greater than 0.87 (27). Participants responded to each item on a 5-point Likert scale, with higher scores indicating higher levels of academic burnout. In this study, Cronbach’s alpha coefficients for the LBS across the three measurement points (T1 through T3) were found to be 0.90, 0.90, and 0.91, respectively. Furthermore, the MacDonald ω coefficients range from 0.91 to 0.91 across T1 to T3.

### 2.3 Data analysis

Data analyses were conducted using SPSS 25.0 and R 4.2.1. Before formal analysis, attrition analyses were conducted to examine whether participants with incomplete follow-up data differed systematically from those who completed all three waves, thereby assessing the plausibility of the Missing at Random (MAR) assumption. Missing data in the RI-CLPMs were subsequently handled in “lavaan” using full information maximum likelihood with robust maximum likelihood estimation (28). Preliminary procedures included descriptive statistics, and Pearson’s correlations. Intraclass correlations (ICCs) were computed to partition variance into between-person and within-person components. To ensure construct comparability over time, we assessed longitudinal measurement invariance by sequentially testing configural, metric (weak), and scalar (strong) models.

To disentangle stable trait differences from within-person dynamics, we employed the RI-CLPM (Hamaker et al., 2015). Model fit was evaluated using standard indices: CFI and TLI > 0.90, and RMSEA and SRMR < 0.08 (29). Following Hamaker et al.’s recommendations regarding parsimony, we determined the optimal model structure by comparing four nested specifications ranging from fully unconstrained to fully stationary (where autoregressive and cross-lagged paths are time-invariant). Model comparisons were based on the Chi-square difference test.

Finally, we examined gender heterogeneity using multigroup RI-CLPM. We compared an unconstrained model (allowing structural paths to vary by gender) against a constrained model (equating autoregressive and cross-lagged coefficients across groups). Subsequently, a chi-square difference test was conducted to compare the fit of the unrestricted model with that of the gender-invariant model.

## 3 Results

### 3.1 Attrition analyses

Attrition analyses indicated no significant differences between participants who complete data on key variables across all three waves (*n* = 4,903) and those who dropped out of the study (*n* = 714) on gender, χ^2(1)^ = 2.44, *p* = 0.12; PMPU, *t* = 1.95, *p* = 0.051, Cohen’s *d* = 0.08; academic burnout, *t* = 0.30, *p* = 0.76, Cohen’s d = 0.01. However, a significant difference was found in age between participants who dropped out (*M* = 19.57, *SD* = 1.84) and those who completed all three assessments (*M* = 19.28, *SD* = 0.67), *t* = 4.12, *p* < 0.001, Cohen’s *d* = 0.21. It is important to note that the statistical significance of this age difference may be driven by the large sample size. Given the small effect size (Cohen’s *d* = 0.21), this difference was considered to lack practical significance for the study’s conclusions.

### 3.2 Preliminary analysis

**Table 1** summarizes the descriptive statistics and correlations. All variables followed a normal distribution (skewness < |2.0| and kurtosis < |7.0|) and were significantly correlated (p < 0.001). Longitudinally, both PMPU (*r* = 0.49 - 0.60) and academic burnout (*r* = 0.59 - 0.69) showed moderate-to-strong temporal stability. Cross-sectional associations were consistently positive (*r* = 0.51- 0.54).

**Table 1:** Descriptive statistics and correlations among variables over time.

Variables	1	2	3	4	5	6
1. PMPU T1	1					
2.PMPU T2	0.60^***^	1				
3.PMPU T3	0.49^***^	0.59^***^	1			
4.Academic Burnout T1	0.54^***^	0.42^***^	0.37^***^	1		
5.Academic Burnout T2	0.41^***^	0.51^***^	0.41^***^	0.69^***^	1	
6.Academic Burnout T3	0.36^***^	0.42^***^	0.53^***^	0.59^***^	0.69^***^	1
*M*	37.58	36.08	34.62	52.74	54.14	53.57
*SD*	11.27	11.69	12.55	11.08	11.25	11.71
Skewness	0.62	0.57	0.64	-0.40	-0.47	-0.60
Kurtosis	0.26	0.02	-0.01	0.33	0.46	0.56

^***^ *p* < 0.001; skewness < |2.0| and kurtosis < |7.0| PMPU, Problematic mobile phone use.

The ICCs were 0.55 for PMPU and 0.65 for academic burnout, indicating that between-person differences account for 55% and 65% of the total variance, respectively. Crucially, the remaining within-person fluctuations (35% - 45%) provided sufficient variance to justify the application of the RI-CLPM to investigate dynamic changes over time.

### 3.3 Measurement invariance

To ensure meaningful longitudinal comparisons, we examined measurement invariance across time points (**Table 2**). Results supported longitudinal metric (weak) invariance, as changes in fit indices were negligible (Δ*CFI*, Δ*TLI* ≤ 0.01, Δ*RMSEA* ≤ 0.015). This confirms that factor loadings remained stable over the three waves, satisfying the prerequisite for the subsequent RI-CLPM analysis.

**Table 2:** Measurement scale invariance over time.

Model	*CFI*	*TLI*	*RMSEA* [90%CI]	*SRMR*	Δ*CFI*	Δ*TLI*	Δ*RMSEA*
PMPU							
Model 1: Configural Invariance	0.970	0.959	0.053 [0.052, 0.055]	0.031			
**Model 2: Weak Invariance**	**0.968**	**0.960**	**0.053 [0.051, 0.054]**	**0.037**	**0.002**	**0.001**	**< 0.001**
Model 3: Strong Invariance	0.952	0.945	0.062 [0.061, 0.063]	0.045	0.018	0.014	0.009
Academic burnout							
Model 1: Configural Invariance	0.948	0.925	0.056 [0.055, 0.057]	0.038			
**Model 2: Weak Invariance**	**0.947**	**0.930**	**0.054 [0.053, 0.055]**	**0.040**	**0.001**	**0.005**	**0.002**
Model 3: Strong Invariance	0.921	0.904	0.064 [0.063, 0.065]	0.049	0.027	0.021	0.008

Bold indicates final selected model. PMPU, Problematic mobile phone use.

### 3.4 Model fit and test of time invariance

The model fits and the results of the chi-square tests for constrained and unconstrained RI-CLPMs are reported in **Table 3**. The unconstrained baseline model (Model 1) demonstrated an excellent fit to the data (*χ*^2^/*df* = 0.894, *CFI* = 0.999, *TLI* = 0.999, *RMSEA* < 0.001, *SRMR* = 0.002). Moreover, the model fits with autoregressive effects (Model 2), cross-lagged effects (Model 3), and both autoregressive and cross-lagged effects (Model 4) constrained to be equal over time all showed excellent fit and were not significantly different from the baseline model(*p* > 0.05). Consequently, based on the principle of parsimony, the RI-CLPM with time-invariant autoregressive and cross-lagged effects (Model 4) was selected as the final RI-CLPM, in which the autoregressive and cross-lagged effects were constrained to be equal across the two adjacent intervals.

**Table 3:** Model fit and model comparisons for RI-CLPM.

Model	*χ*^2^/*df*	*CFI*	*TLI*	*RMSEA*	*SRMR*	Comparison model	*P*
M1: Baseline model (unconstrained model)	0.894	0.999	0.999	< 0.001	0.002		
M2: Model with autoregressive paths fixed to be time-invariant	0.491	0.999	0.999	< 0.001	0.003	M2 vs M1	0.800
M3: Model with cross-lagged paths fixed to be time-invariant	1.275	0.999	0.999	0.007	0.005	M3 vs M1	0.240
**M4: Model with autoregressive and cross-lagged paths fixed to be time-invariant**	**1.037**	**0.999**	**0.999**	**0.003**	**0.005**	**M4 vs M1**	**0.453**

Bold indicates final selected model.

### 3.5 Random intercept cross-lagged panel models

**Figure 2:**
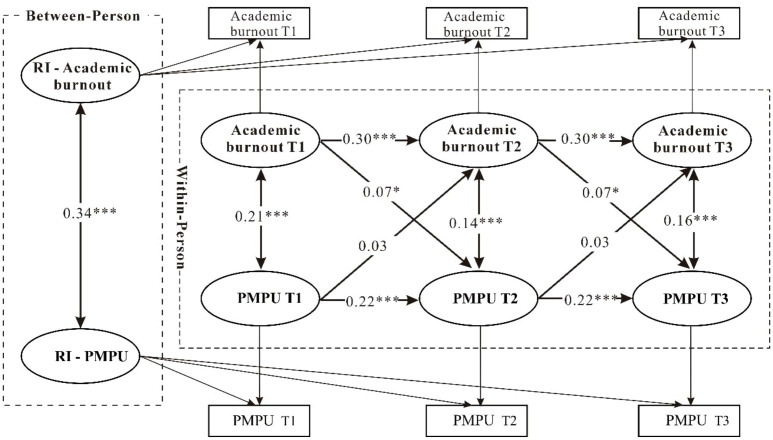
Standardized path coefficients of the final RI-CLPM. ^*^p < 0.05, ^***^p < 0.001. PMPU, Problematic mobile phone use.

**Figure 2** displays the standardized path coefficients of the final RI-CLPM (see **Table 4** for more details). At the between-person level, the random intercepts of PMPU and academic burnout were significantly correlated (*β* = 0.34, *p* < 0.001), indicating that there were significant between-person effects linking the stable variances between them.

**Table 4:** Estimated parameters of the cross-lagged panel models.

Paths	*β*	*se*	*z*	*p*
Between - Person				
Academic Burnout ↔ PMPU	0.34	0.02	18.42	< 0.001
Relevant covariances				
Academic Burnout T1 ↔ PMPU T1	0.21	0.02	12.35	< 0.001
Academic Burnout T2 ↔ PMPU T2	0.14	0.01	10.84	< 0.001
Academic Burnout T3 ↔ PMPU T3	0.16	0.01	14.65	< 0.001
Autoregressive paths				
Academic Burnout T1 → Academic Burnout T2	0.30	0.04	8.43	< 0.001
Academic Burnout T2 → Academic Burnout T3	0.30	0.04	8.43	< 0.001
PMPU T1 → PMPU T2	0.22	0.04	7.25	< 0.001
PMPU T2 → PMPU T3	0.22	0.04	7.25	< 0.001
Cross-lagged paths				
Academic Burnout T1 → PMPU T2	0.07	0.03	2.56	0.01
Academic Burnout T2 → PMPU T3	0.07	0.03	2.56	0.01
PMPU T1 → Academic Burnout T2	0.03	0.02	1.74	0.08
PMPU T2 → Academic Burnout T3	0.03	0.02	1.74	0.08

PMPU, Problematic mobile phone use. Directional arrows indicate autoregressive and cross-lagged paths; Bidirectional arrows indicate covariances or between-person associations.

At the within person level, the autoregressive path revealed that deviations in academic burnout at T2 were predicted by deviations in academic burnout at T1, academic burnout at T3 were predicted by deviations in academic burnout at T2 (*β* = 0.30, s*e* = 0.04, *p* < 0.001). Similarly, PMPU at T + 1 was predicted by deviations of PMPU at T (*β* = 0.22, s*e* = 0.04, *p* < 0.001).

Examining the cross-lagged paths, the RI-CLPM showed that the paths from academic burnout at T to PMPU at T + 1 were statistically significant (*β* = 0.07, s*e* = 0.03, *p* = 0.01). However, the paths from PMPU at T to academic burnout at T + 1 were not significant (*β* = 0.03, s*e* = 0.02, *p* = 0.08).

### 3.6 Gender invariance of the RI-CLPM

Finally, a multiple group RI-CLPM was employed to assess the invariance of the model across gender. First, we fitted a cross-gender RI-CLPM with autoregressive and cross-lagged paths fixed to be time-invariant within gender (*χ*^2^/*df* = 2.40, CFI = 0.99, TLI = 0.99, RMSEA = 0.021, SRMR = 0.009). Subsequently, we constrained the autoregressive and cross-lagged paths fixed to be time-invariant as invariance across the gender (*χ*^2^/*df* = 1.65, CFI = 0.99, TLI = 0.99, RMSEA = 0.016, SRMR = 0.010). The chi-square difference test for the two nested models was (Δ*χ*^2^ = 2.11, *p* = 0.72), indicting the model invariance across gender.

## 4 Discussion

The present study employed a three-wave Random Intercept Cross-Lagged Panel Model (RI-CLPM) to disentangle the longitudinal dynamics between academic burnout and PMPU among Chinese freshmen. Moving beyond the stable associations observed at the between-person level, our analysis revealed a significant longitudinal predictive association at within-person level. Specifically, deviations in academic burnout consistently predicted subsequent increases in PMPU over time. Conversely, the reverse path from PMPU to academic burnout was not statistically significant throughout the study period. Furthermore, this dynamic mechanism was found to be invariant across gender. Collectively, these findings suggest that PMPU reflect a maladaptive coping response associated with academic stress.

### 4.1 Between-person association between academic burnout and PMPU

Consistent with Hypothesis 1, our RI-CLPM analysis revealed a significant positive correlation between the random intercepts of academic burnout and PMPU. This finding indicates that, at the trait level, students who generally experience higher levels of academic burnout throughout their freshman year are also more likely to exhibit severe symptoms of mobile phone addiction. This result aligns with extensive cross-sectional literature documenting the robust link between academic stress and addictive behaviors (17, 30, 31). It suggests that there may be shared stable vulnerability factors, such as personality traits (e.g., neuroticism) (32) or chronic environmental stressors (30), maladaptive coping styles (33), or broader self-regulatory vulnerabilities (34)may simultaneously predispose individuals to both higher burnout and greater susceptibility to PMPU. From this perspective, the observed correlation captures a shared vulnerability structure that operates across individuals, rather than a dynamic process unfolding within individuals over time.

### 4.2 The within-person dynamics: evidence for a predominantly burnout-to-PMPU pathway

Our RI-CLPM analysis provided robust support for Hypothesis 2, revealing that deviations in academic burnout significantly positively predicted subsequent deviations in PMPU at the within-person level. This finding offers longitudinal empirical evidence for the Compensatory Internet Use Theory (11) within a dynamic resource framework. Consistent with prior research linking academic stress to addictive behaviors (35), our results confirm that burnout represents a critical state of resource depletion, characterized by the exhaustion of emotional and cognitive energy. Driven by the motivation to halt further loss, students resort to the mobile phone as a low-cost coping tool for instant gratification and psychological detachment (30). Thus, PMPU in present study reflect a maladaptive compensatory coping response associated with academic exhaustion. Conversely, contrary to the typical expectations of a reciprocal relationship and the Displacement Hypothesis (36), our results did not support a significant reverse pathway from PMPU to subsequent academic burnout during the present study period at the within-person level. From a Job Demands-Resources (JD-R) perspective (37), academic burnout appears driven more by external structural stressors than individual behavioral habits. Since burnout is primarily triggered by objective high demands (e.g., curriculum loads), PMPU does not alter the actual academic pressure imposed by the university. Consequently, burnout remains tethered to the environmental context rather than daily fluctuations in leisure screen time. Furthermore, the transition from high-stakes examination culture to university autonomy likely creates a psychological contrast effect. For freshmen, the subjective appraisal of academic burden—even amidst excessive phone use—may remain low relative to their pre-university intensity, thereby buffering the immediate impact of time displacement. It is also plausible that the adverse consequences of PMPU operate through a delayed feedback loop, requiring a duration longer than the current one-year scope to fully materialize and exacerbate exhaustion.

### 4.4 Gender invariance: a generalized coping mechanism

Finally, our multi-group analysis supported Hypothesis 3, revealing that the longitudinal associations between academic burnout and PMPU were invariant across gender. This finding suggests that the depletion-compensation mechanism identified in this study operates universally among Chinese freshmen, transcending gender distinctions. While prior cross-sectional studies have often noted gender differences in the prevalence (38, 39)or content of mobile phone use (40), our RI-CLPM results indicate that the underlying functional driver remains identical. Regardless of gender, the drive to conserve resources by retreating into a low-effort digital environment appears to be a fundamental response to stress during this transitional period. Thus, the resource depletion pathway emerges as a fundamental human response to environmental demands during the university transition, rather than a gender-specific phenomenon.

### 4.5 Practical implications

The findings of the present study have significant practical implications for university educators and mental health practitioners. Crucially, the observed longitudinal association between academic burnout and subsequent PMPU suggests that PMPU may partly reflect responses to academic resource depletion among freshmen. Interventions focusing exclusively on reducing mobile phone use without addressing academic burnout may have limited effectiveness, or could potentially drive students toward other maladaptive coping mechanisms. Therefore, interventions should consider addressing academic stress as a potential intervention target, rather than focusing solely on the symptom of digital usage.

Furthermore, since the mechanism is driven by resource depletion, universities should provide resources that facilitate recovery. Given that students use phones as a low-effort recovery strategy, educators should introduce healthier, low-cost alternatives for psychological detachment and relaxation. For example, integrating stress management training and time-management workshops into freshman orientation programs could help students navigate the transition from high school to university more effectively. Since our multi-group analysis confirmed that this mechanism is gender-invariant, these stress-reduction programs can be implemented universally without the need for gender-segregated protocols.

### 4.6 Limitation and future directions

Several limitations warrant consideration when interpreting these findings. First, although focusing exclusively on Chinese freshmen minimized heterogeneity during the post-Gaokao transition, the use of convenience sampling from four universities in a single Chinese province limits the ecological validity and generalizability of the findings. Future research should use more geographically and institutionally diverse samples to investigate whether this burnout-driven mechanism persists in populations subject to different structural academic pressures. Second, the reliance on self-report measures introduces potential common method variance. Future inquiries should triangulate subjective assessments with objective indicators, such as digital screen-time logs or official academic transcripts. Third, although longitudinal metric invariance was supported across the three waves, scalar invariance showed comparatively weaker fit. Therefore, the present findings are more appropriately interpreted as evidence regarding longitudinal structural associations and within-person dynamic processes rather than absolute latent mean-level changes across time. Fourth, current study did not include a broader set of clinical control variables such as depressive symptoms, anxiety symptoms, sleep quality. Future studies should incorporate these variables to better isolate the specific longitudinal association between academic burnout and PMPU. Finally, the six-month temporal interval may have influenced the null result regarding the reverse displacement path. The cumulative negative impact of PMPU on academic exhaustion likely operates over a more protracted timeline than the one-year duration captured in this study. Future scholarship should employ diverse temporal designs, including intensive longitudinal methods or multi-year cohorts, to fully map the dynamic boundaries and potential latency effects of these reciprocal relationships.

## 5 Conclusion

The present study employed a rigorous RI-CLPM approach to elucidate the dynamic interplay between academic burnout and PMPU among Chinese university freshmen. By distinguishing within-person fluctuations from stable trait differences, our findings help clarify the temporal ordering of this relationship, showing that higher academic burnout prospectively predicts subsequent increases in PMPU at the within-person level. Specifically, PMPU reflect a maladaptive coping response associated with resource depletion. Notably, this unidirectional coping pathway remains consistent across genders. These results emphasize that, for university freshmen, excessive mobile phone use acts primarily as a compensatory mechanism for academic resource depletion rather than a contributor to academic exhaustion. Consequently, intervention strategies should prioritize alleviating academic stress and restoring student well-being, rather than focusing solely on restricting digital behavior.

## Data Availability

The datasets presented in this article are not readily available because contains specific information about the students. Requests to access the datasets should be directed to Li Chen, psychologychenli@163.com.
